# Self-Transcendence: Association with Spirituality in an Italian Sample of Terminal Cancer Patients

**DOI:** 10.3390/bs13070559

**Published:** 2023-07-05

**Authors:** Andrea Bovero, Sara Pesce, Rossana Botto, Valentina Tesio, Ada Ghiggia

**Affiliations:** 1 Clinical Psychology Unit, AOU Città della Salute e della Scienza, Corso Bramante 88, 10126 Turin, Italy; 2 Department of Neuroscience, University of Turin, AOU Città della Salute e della Scienza, Corso Bramante 88, 10126 Turin, Italy; 3 Department of Psychology, University of Turin, Via Verdi 10, 10124 Turin, Italy; valentina.tesio@unito.it; 4 Department of Life Sciences, University of Trieste, Via Edoardo Weiss 21, 34128 Trieste, Italy

**Keywords:** spirituality, self-transcendence trait, psychooncology, end of life, cancer

## Abstract

Terminally ill cancer patients often experience demoralization and loss of dignity, which undermines their spiritual wellbeing, which could, however, be supported by the presence of other factors such as self-transcendence and religious coping strategies. To assess self-transcendence and religious coping strategies and how they influence spirituality, we studied 141 end-stage cancer patients (64.3% male; mean age 68.6 ± 14.6) with a Karnofsky Performance Status ≤ 50 and a life expectancy ≤ 4 months using the Self-Transcendence Scale, the Demoralization Scale, the Functional Assessment of Chronic Illness Therapy—Spiritual Wellbeing (FACIT-Sp-12), the Brief Religious COPE, and the Patient Dignity Inventory. To understand the effects of these variables on spirituality, hierarchical multiple regression was performed on FACIT-Sp-12. The final model predicted 67% of the variance in spiritual wellbeing. Demoralization was the strongest influencing factor (β = −0.727, *p* < 0.001), followed by self-transcendence (β = 0.256, *p* < 0.001), and positive religious coping (β = 0.148, *p* < 0.05). This study suggests that self-transcendence and positive religious coping may be protective factors for spirituality in terminal cancer patients. These factors should be considered in treatment to promote spiritual wellbeing and improve patients’ quality of life at the end of life.

## 1. Introduction

Palliative care is defined by the World Health Organization as “the comprehensive care of patients whose illness does not respond to curative treatment”. The goal of palliative care is to achieve the best possible quality of life for patients and their families. Therefore, treatment includes complete control of pain and other symptoms, psychological and social distress, and, last but not least, spiritual problems. 

Spirituality involves believing in something greater, finding meaning in something that cannot be seen with one’s own eyes [1]. It may be important for good psychological development and adaptation to the disease, as it removes the sense of impotence that would otherwise make life hopeless. The spiritual dimension seems to alleviate patients’ psychological distress by mediating their attachment style [2]. Spirituality is not only about religion. Moreover, religion can also lead to maladjustment when the person loses sight of reality and waits trustingly for divine instructions. Previous studies have shown that positive religious coping strategies are protective factors for psychological distress and, more broadly, for quality of life in patients with advanced disease [3]. Moreover, positive religious coping strategies have been shown to improve the quality of life of patients with advanced cancer [4,5] and alleviate feelings of distress [6,7].

Spirituality and positive religious coping strategies play an important role in the pursuit of meaning by redefining a stressful situation as an opportunity for spiritual growth [8]. This perception became part of people’s structure of meaning to cope with life’s difficulties [9]. Since the meaning of life is a feeling that can be fostered through self-transcendence [10], this trait could be considered a protective factor for psychological distress. 

Furthermore, it can be argued that spirituality could be considered a concept of self-transcendence, as this trait is associated with spiritual ideas [11]. According to Cloninger’s model of temperament and character [12], self-transcendence is a character dimension that refers to the ability to perceive oneself as an integral part of the world and everything that surrounds it. Self-transcendence affords individuals the ability to see themselves as spiritually connected to all that surrounds them. This perspective is described by Cloninger as “acceptance, identification, or spiritual union with nature and its source” [12].

The concept of self-transcendence has been studied over the years in the context of spirituality [13,14], meaning of life [15], and quality of life in palliative care [16]. According to Reed’s theory [17], self-transcendence increases self-awareness and introspection [10] and may promote spiritual wellbeing by providing a sense of purpose, acceptance, and self-efficacy [18]. In addition, self-transcendence has been studied within the concept of religious coping strategies [19]. Pargament and colleagues [20] identified two patterns of religious coping: the positive, which consists of religious coping methods such as seeking spiritual support and connecting with God, and the negative, which is characterized by spiritual dissatisfaction and distrust of God. Both religious coping strategies, especially the positive, are often used to cope with adverse events and can help people find meaning and purpose [20]. 

To date, spiritual wellbeing, self-transcendence, and religious coping have been studied in relation to populations of older adults and people with terminal illnesses such as cancer [2,4,21,22,23,24,25,26]. Ill patients are exposed to a very stressful situation due to their physical and psychological symptoms and therefore often suffer from mental health problems [27]. The most common symptoms in people with a cancer diagnosis include anxiety and/or depression [28,29,30], but especially demoralization [31,32] and loss of dignity [33,34]. 

Based on these findings, the present study aimed to shed more light on the aspects that may be protective or risky for spiritual wellbeing in a group of terminal cancer patients. Specifically, first, we wanted to observe the presence of self-transcendence and religious coping strategies in the most fragile and distressing phase of life. Second, we wanted to observe how these variables, along with risk factors such as demoralization and loss of dignity, may influence spirituality at the end of life.

## 2. Materials and Methods

### 2.1. Setting and Sample

The final sample consisted of 141 hospitalized patients recruited between April 2019 and March 2020 at Molinette Hospital “Città della Salute e della Scienza” (79 (55.2%) patients) and at Hospice “Valletta” (62 (43.4%) patients) (Turin, Italy).

All participants had a cancer diagnosis in palliative care, with a life expectancy of only a few weeks. Under regional law, patients are admitted to palliative care if they are in an advanced stage of disease (terminal phase) for which curative treatment is not possible or appropriate, and if they have an unfavorable/poor prognosis, with an expected life expectancy of 4 months or less and a Karnofsky Performance Status (KPS) [35,36] of 50 or less (Piedmont Regional Legislative Decree No. 45/2002 and National Law on Palliative Care and Pain Management No. 38/2010). Patients who met the inclusion criteria were asked to participate in the study and were consecutively included if they consented to the study. All participants provided written informed consent, and the present study was approved by the Ethics Committee of the institution (Procedure n° CS2/1178) in accordance with the Declaration of Helsinki. 

Palliative care physicians recorded the sociodemographic (age, sex, marital status, education, and occupation) and clinical data of each patient. Psychological data were collected by psychooncologists who provide psychological counseling in hospitals and hospices and offer psychological interventions to patients and caregivers. A bedside interview was conducted, and questionnaires listed in the Instruments section were distributed to patients who agreed to participate in this study. Patients who were independent were asked to complete the questionnaires in their own time. In cases where physical conditions did not permit complete independence and completion of the questionnaires, the researcher assisted the patient by, for example, reading aloud or spreading the data collection over several days.

### 2.2. Instruments 

#### 2.2.1. Clinical Variables and Spiritual Well-Being

The Karnofsky Performance Status (KPS) is a physician-rated index of physical performance often used in studies of cancer patients [35,36,37]. A higher score (range 0–100) means that the patient is able to perform daily activities.

The Functional Assessment of Chronic Illness Therapy—Spiritual Wellbeing (FACIT-Sp-12) is a 12-item instrument designed to assess spiritual wellbeing in cancer patients [38]. Each item is scored on a five-point Likert scale (from “not at all” to “very much”), with total scores ranging from 0 to 48: the higher the total score, the higher the level of spirituality. Originally, the instrument contained three subscales expressed in different factors [39]: meaning, peace, and faith. More recently, a new three-factor model has been proposed [40], in which the first factor is called meaning and consists of 4 items, whereas the second and third factors are called peace and faith, respectively, and consist of 2 items. In our sample, the internal reliability of FACIT-Sp-12 estimated using Cronbach’s alpha was 0.85.

#### 2.2.2. Psychological Variables

The Temperament and Character Inventory Revised (TCI-R) [41,42] was developed to assess the temperament and character domains according to Cloninger’s theory. In the revised version of the instrument, the author introduced three dimensions of character [43]: self-control (SD), cooperativeness (C), and self-transcendence (ST). Given the purpose of our study, which focuses on the analysis of self-transcendence, we chose to use only the ST scale in our sample, as suggested by Garcia-Romeu [44]. The ST scale consists of 21 items rated on a five-point Likert scale from one (absolutely wrong) to five (absolutely right). The construct of self-transcendence was described by Cloninger [12] as a three-stage developmental sequence measured using three corresponding subscales: self-forgetful vs. self-aware experience (ST1), transpersonal identification vs. self-isolation (ST2), and spiritual acceptance vs. rational materialism (ST3). In our sample, the internal reliability of the Self-Transcendence Scale estimated using Cronbach’s alpha was 0.92.

The Brief Religious COPE (Brief RCOPE) [45,46] is a 14-item instrument used to evaluate two modes of cancer-related religious coping: positive and negative. The instrument consists of 14 items rated on a Likert scale from one (not at all) to four (very strongly). It is divided into two subscales; the first includes items from one to seven and measures positive religious coping (e.g., “looked for a stronger connection with God”), while the second consists of items from 7 to 14 and measures negative religious coping (e.g., “wondered whether God had abandoned me”). 

The Patient Dignity Inventory (PDI) is a 25-item scale that measures distress related to dignity by examining the patient’s level of difficulty with physical, psychological, and social problems on a 5-point Likert scale ranging from 1 (no problem) to 5 (it is a distressing problem) [47,48]. The instrument aims to capture the multiple causes of distress (physical, functional, psychological, existential, and spiritual problems), and it includes the following five subscales: psychological distress, social support, physical symptoms and dependence, existential distress, and loss of meaning and purpose [49]. In our sample, the internal reliability of the PDI scale estimated using Cronbach’s alpha was 0.93.

The Demoralization Scale (DS) is a 24-item scale assessing patient demoralization, with a 5-point Likert scale ranging from 0 (never) to 4 (always) describing the frequency of occurrence of each item [50,51]. The instrument consists of five subscales expressing the dimensions of demoralization examined in the questionnaire: loss of meaning and purpose, dysphoria, discouragement, helplessness, and sense of failure [50,51]. In our sample, the internal reliability of the Demoralization Scale estimated using Cronbach’s alpha was 0.93.

### 2.3. Statistical Analyses 

Asymmetry and kurtosis were used to prove the normal univariate distribution of the data. Means (SD) and frequencies were reported as descriptive analyses. Student’s t tests for two independent samples and χ2 tests were performed to determine whether there were differences between gender (female and male) in clinical, sociodemographic, or psychological variables. Furthermore, two preliminary one-way ANOVAs were conducted to determine whether psychological variables differed between groups with different levels of education and marital status. Relationships among variables (demographic, clinical, and psychological) were analyzed using Pearson’s bivariate correlation. Hierarchical linear regression was performed to examine whether demoralization, positive and negative religious coping, and self-transcendence significantly contributed to the explanation of spirituality, with FACIT-Sp-12 total score serving as the dependent variable. Linearity and homoscedasticity were tested using partial regression plots and a plot of studentized residuals against predicted values, while independence of residuals was tested using the Durbin–Watson statistic. The P–P plot was used to test the normal distribution of the residuals. Regarding multicollinearity, tolerance and variance inflation factors were used. There were no studentized deleted residuals greater than ±3 standard deviations, no leverage values greater than 0.2, and no Cook’s Distance values greater than 1. A 2-tailed test with a value of 0.05 was used for all statistical analyses.

Statistical analysis was performed using SPSS Statistics version 26.0.0 software for Mac (IBM Corp. Armonk, New York, NY, USA). 

Sample size was calculated using G*Power software (version 3.1). For hierarchical regression analysis considering 1 dependent variable (FACIT-Sp-12 total score) and 5 independent variables (gender, demoralization, loss of dignity, positive coping, and self-transcendence), effect size F^2^ = 0.15; α = 0.05, and power of 0.95; the required sample size was 138.

## 3. Results

### 3.1. Sociodemographic, Clinical, and Psychological Characteristics

Sociodemographic, religious practices and clinical data are shown in Table 1. The mean age is over 65 years (mean 68.5 ± 14.64), and in terms of gender, the sample is predominantly male (64.3%). Regarding the type of disease, lung cancer was the most common. Significant differences between sex were found in marital status (χ^2^(4) = 14.7, *p* = 0.005) and religious practice (χ^2^(1) = 6.5, *p* = 0.011).

The psychological variables (mean and standard deviation) are shown in Table 2. The results of the ANOVAs showed that the differences between both educational levels and marital status were not statistically significant for any of the psychological variables. So, education and marital status were not included in the subsequent analyses. Significant differences between sex were found in FACIT-Sp-12 total score (female vs. men, mean SD: 24.0 ± 8.4 vs. 21.2 ± 6.1; t(76) = −2.06, *p* = 0.02), and in Positive Religious Coping (female vs. men, mean SD: 15.1 ± 5.6 vs. 11.4 ± 4.9; t(137) = −4.01, *p* < 0.001).

### 3.2. Correlational Analysis

The results of the bivariate correlations between spiritual wellbeing and psychological variables are presented in Table 3 (r values are reported in table). Age was not correlated with any of the psychological scales. A significant negative correlation was found between demoralization (DS-IT) and all the spirituality scores (FACIT-Sp-12; all *p* ≤ 0.01). Regarding the PDI total score, we found a significant and negative correlation with all the spirituality scores (*p* < 0.05). Positive and significant correlations were also found between the positive religious coping score and spirituality and faith subscales, while the negative religious coping score did not correlate with spirituality (*p* > 0.05). 

For the Self-Transcendence scale, a positive and significant correlation was found between the total score of FACIT-Sp-12 (r = 0.483, *p* < 0.01) and peace (r = 0.294, *p* = 0.294) and Faith subscales (r = 0.607, *p* < 0.01). On the other hand, no significant correlation was found between the subscale FACIT-Sp-12 meaning and the Self-Transcendence Scale (r = 0.136, *p* = 0.108). To investigate any differences between sex, the correlations for females and males were analyzed separately. The results showed no differences between female and male, except for the correlation between the total PDI score and FACIT-Sp-12, which was not statistically significant for either sex.

### 3.3. Regression Analyses

Hierarchical multiple regression was conducted to examine whether the inclusion of the self-transcendence trait, in addition to loss of dignity, positive religious coping, and demoralization resulted in significantly higher variance in spirituality. As a result of the preliminary correlational analysis, the following five blocks of variables were introduced sequentially: (1) gender; (2) demoralization; (3) sense of dignity; (4) positive religious coping; (5) self-transcendence trait (see Table 4 for the regression model). The addition of demoralization to the model (Step 2) resulted in a statistically significant increase in R^2^ by 0.52, F(1, 136) = 55.11, *p* < 0.001, indicating a negative contribution of demoralization. In the third model (step 3), loss of dignity (PDI) was added. Although the effect of demoralization remained high, PDI also proved to be a statistically significant predictor.

The full model (Step 5) predicting spirituality was statistically significant, with R^2^ of 0.67, F(5, 133) = 53.36, *p* < 0.001. Demoralization remained the most significant and negative predictor (β = −0.727, *p* < 0.001). On the other hand, self-transcendence and positive religious coping emerged as significant positive predictors of spirituality (TCI-ST: β = 0.256, *p* < 0.001; positive religious coping: β = 0.148, *p* = 0.021). Loss of dignity was also a significant predictor of spirituality, although with a lower β (β = 0.148, *p* = 0.045).

## 4. Discussion

The aim of the present study was to investigate the relationship between self-transcendence, positive and negative religious coping, demoralization, and dignity in a sample of terminal cancer patients and to determine their relationship with spiritual wellbeing. Correlational analysis showed a significant and positive relationship between self-transcendence and spiritual wellbeing [26,52]. These results support information in the literature indicating that self-transcendence can promote spiritual wellbeing: self-transcendent traits seem to favor the ability to find meaning in social and spiritual life, thus increasing spiritual wellbeing [17]. In addition, our data highlighted that self-transcendence was significantly associated with positive religious coping strategies. Although limited data are available in the literature, these findings are consistent with previous studies showing low association between negative religious coping strategies and self-transcendence [19]. In support of these findings, regression analysis indicates that self-transcendence and positive religious coping strategies are predictors of spiritual wellbeing. This result could be due to the fact that the two factors studied have in common the concept of the search for meaning. Indeed, it is known that the presence of self-transcendence as a character trait implies a search for meaning in daily life [11], and this is closely related to positive religious coping. Indeed, positive strategies accompany actions that help individuals find meaning in their relationship with God [20]. Therefore, the search for meaning fostered by self-transcendence and positive religious coping strategies may promote spiritual wellbeing [53]. 

Moreover, correlational analysis showed a significant negative relationship between the self-transcendence scale and the demoralization scale. These results support the idea that the sense of purpose promoted by self-transcendence can reduce psychological distress in patients at the end of life [16]. In particular, feelings of discouragement and failure can be alleviated by promoting a sense of meaning in life, which may help patients reduce psychological distress [54].

In addition, the demoralization scale showed a negative relationship with spiritual wellbeing, which was confirmed by the regression analysis in which demoralization appeared as the main significant negative predictor of spiritual wellbeing. The regression analysis also showed that self-transcendence significantly predicted spiritual wellbeing. Positive religious coping strategies are often used to cope with adverse events and can help people find meaning and purpose [20]. Cancer patients face a very stressful situation due to their physical and psychological symptoms and therefore often suffer from psychological problems [27]. Spiritual wellbeing, self-transcendence, and positive religious coping may therefore act as protective factors.

Further research is encouraged to investigate the role of these variables in reducing psychological distress and promoting wellbeing. Further studies should also delve into the relationship between loss of dignity, demoralization, and spiritual wellbeing. Indeed, the correlation analyses showed a significant and negative relationship between loss of dignity and spiritual wellbeing. However, the regression analysis showed a doubtful result, where demoralization seems to play a crucial role in elucidating this relationship. Spiritual wellbeing has been shown to be related to the presence of character traits associated with self-transcendence [55]. This finding clearly implies the need to study self-transcendence in patients and to promote its structuring also through positive coping strategies that can support a spiritual approach in patients. These results could also enlighten about the best intervention to treat the patient’s suffering, namely a more spiritual path that can lead to the psychological wellbeing of the person. Moreover, the presence of traits of self-transcendence may enable the patient to try, together with the physician, to find meaning in their illness at the end of life. The evolution of psychotherapies in palliative care has led to the development of strategies focused on spirituality, based on the patient’s need for answers not only to physical, mental, or social needs, but also to the existential questions about the meaning or value of life and spirituality. Palliative care psychotherapists seize the opportunity to encourage patients to find meaning in suffering in the face of death. This meaning-making process can be generative for patients: several studies confirm the use of validated psychotherapies to restore meaning. These include Victor Frankl’s logotherapy [56,57] and Chochinov’s dignity therapy [58,59]. Thus, psychotherapy helps to analyze the patient’s meaning of life from a psychological point of view, performing activities that allow for defining the trace of self that the patient wants to leave to the next generation, in order to promote the connection that allows giving meaning to one’s life. 

In addition to psychotherapists, other professions are also concerned with spirituality, such as healthcare chaplains (albeit with different methods, interpretations, and goals), and previous work has addressed this topic [60]. Nevertheless, our research focuses on psychological aspects and on a psychological perspective. In palliative care, a difficult challenge for the psychotherapist is to awaken social feelings through an empathic understanding of a patient’s suffering and accompanying the person with love and hope until the end of life in a compassionate way [61].

In conclusion, from a clinical perspective, these data are of great importance in clinical and palliative settings, as they could pave the way for new tailored treatments that target patients’ spiritual and psychological wellbeing.

The strengths of this study lie in the analysis of factors such as spirituality and the ability to find meaning in life that have been little studied in the literature, especially in a population of terminally ill people [25]. On the other hand, this study also has some limitations. First, this is a cross-sectional study, which does not allow us to demonstrate a causal relationship between variables or to assess the influence of variables at different stages of the disease. Second, the average age of our sample was quite high. It could be interesting to explore these factors in younger patients with degenerative diseases at the end of life. Third, we did not consider some variables related to the individual’s family dynamics and the presence and qualitative support of the caregiver, especially the religious or spiritual beliefs of the caregiver. This information could better explain self-transcendence and positive religious coping and their role for the patient at the end of life.

## 5. Conclusions

The current study suggests that self-transcendence and positive religious coping strategies may be protective factors in reducing distress in cancer patients in palliative care. 

These factors need to be considered in the treatment of this patient population in order to promote spiritual wellbeing and thus improve patients’ quality of life at the end of life. Indeed, health care providers should consider these aspects when providing personal and medical care. Clinical treatment protocols could be designed to promote the development of characteristics of self-transcendence in patients, help them find meaning even during their illness, and improve psychological and spiritual wellbeing at every stage of the disease.

## Figures and Tables

**Table 1:** Sociodemographic characteristics of the sample.

Characteristics	N = 141	Female (n = 50)	Men (n = 93)
Age, (mean ± SD)	68.55 ± 14.64	67.9 ±16.1	69.9 ± 12.0
Site, n (%)			
Hospice	62 (43.4)	22 (15.8)	40 (28.8)
Hospital	79 (55.2)	26 (18.7)	51 (36.7)
Marital status, n (%)			
Married/cohabitant	93 (65.7)	23 (16.5)	70 (50.3)
Single	22 (16.1)	11 (7.9)	11 (7.9)
Divorced	8 (5.6)	5 (3.6)	3 (2.2)
Widow(er)	16 (11.2)	5 (3.6)	11 (7.9)
Education, n (%)			
Primary school	36 (25.2)	11 (7.9)	25 (18.0)
Middle school	51 (37.1)	22 (15.8)	29 (20.9)
High school	43 (30.8)	14 (10.1)	29 (20.9)
Graduate	9 (6.3)	2 (1.4)	7 (5.0)
Occupation, n (%)			
Unemployed	8 (5.6)	3 (2.2)	5 (3.7)
Employed	22 (15.4)	9 (6.7)	13 (9.7)
Self-employed	11 (8.4)	5 (3.7)	6 (4.5)
Retired	93 (65.7)	29 (21.6)	64 (47.8)
Religion, n (%)			
Catholic	122 (86.0)	43 (31.6)	79 (58.1)
Orthodox	2 (1.4)	2 (1.5)	0 (0)
Atheist	11 (8.4)	2 (1.5)	9 (6.6)
Agnostic	1 (0.7)	0 (0)	1 (0.7)
Religious practice, n (%)			
Prayer	41 (28.7)	21 (15.8)	20 (15.0)
Not a prayer	92 (65.7)	26 (19.5)	66 (49.6)
Tumor site, n (%)			
Lung	32 (23.4)	6 (4.3)	26 (18.6)
Pancreatic	25 (17.7)	9 (6.4)	16 (11.4)
Colorectal	13 (9.2)	6 (4.3)	7 (5.0)
Kidney	13 (9.2)	2 (1.4)	11 (7.9)
Other	57 (40.4)	26 (18.6)	31 (22.1)
KPS (mean ± SD)	39.34 ± 9.76	41.4 ± 8.4	38.2 ± 10.3

Abbreviations. N (%) = number and absolute frequencies; SD = standard deviation; KPS = Karnofsky Performance Status.

**Table 2:** Psychological variables, means ± SD (N = 141).

Variables	N = 141	Female (n = 50)	Men (n = 91)
FACIT-Sp-12			
Total score	22.1 ± 7.1	24.0 ± 8.4	21.2 ± 6.1
Meaning	11.5 ± 2.6		
Peace	6.6 ± 3.2		
Faith	4.1 ± 3.5		
Demoralization Scale			
Total score	37.8 ± 14.2	36.2 ± 13.9	38.5 ±14.4
Loss of meaning and purpose	5.5 ± 4.4		
Dysphoria	6.5 ± 3.2		
Disheartenment	13.0 ± 4.3		
Helplessness	6.3 ± 3.3		
Sense of failure	6.5 ± 2.2		
Patient Dignity Inventory			
Total score	61.6 ± 16.0	59.4 ± 16.8	62.6 ± 15.5
Psychological distress	17.3 ± 5.1		
Social support	4.4 ± 2.0		
Physical symptoms and dependency	12.0 ± 3.7		
Existential distress	19.7 ± 5.9		
Loss of purpose and meaning	8.2 ± 3.3		
Brief RCOPE			
Positive religious coping	12.7 ± 5.4	15.1 ± 5.6	11.4 ± 4.9
Negative religious coping	10.3 ± 3.3	10.8 ± 3.7	10.0 ± 3.1
TCI self-transcendence			
Total score	67.6 ± 17.3	71.6 ± 17.8	65.6 ± 16.7
ST1	25.5 ± 7.6		
ST2	19.8 ± 5.8		
ST3	24.3 ± 7.0		

Abbreviations. FACIT-Sp-12: Functional Assessment of Chronic Illness Therapy—Spiritual Wellbeing Scale; TCI = Temperament and Character Inventory Revised; ST1 = self-forgetful vs. self-conscious experience; ST2 = transpersonal identification vs. self-isolation; ST3 = spiritual acceptance vs. rational materialism.

**Table 3:** Pearson’s correlations among spirituality (FACIT-Sp-12), age, and psychological variables.

	Age	DS-IT	PDI	Positive Religious Coping	Negative Religious Coping	Self-Transcendence
FACIT-Sp-12 total score	−0.004	−0.713 *	−0.288 *	0.349 *	−0.096	0.483 *
FACIT-Sp-12 meaning	−0.028	−0.664 *	−0.186 *	−0.048	−0.068	0.136
FACIT-Sp-12 peace	0.063	−0.713 *	−0.434 *	0.124	−0.119	0.294 *
FACIT-Sp-12 faith	−0.046	−0.303 *	−0.049	0.628 *	−0.036	0.607 *

Abbreviations. FACIT-Sp-12: Functional Assessment of Chronic Illness Therapy—Spiritual Wellbeing Scale; DS-IT: Demoralization Scale, Italian Version; PDI: Patient Dignity Inventory. * *p*-value ≤ 0.01.

**Table 4:** Hierarchical multiple regression with spirituality (FACIT-Sp-12 total score) as dependent variable.

Predictor	B	β	t	R^2^	ΔR^2^	F
Step 1				0.04	0.04	5.06 *
Gender	2.80	0.189	2.25 *			
Step 2				0.53	0.49	75.63 **
Gender	2.01	0.135	2.287 *			
DS-IT	−0.352	−0.703	−11.876 **			
Step 3				0.55	0.02	55.11 **
Gender	2.157	0.145	2.505 *			
DS-IT	−0.405	−0.810	−11.514 **			
PDI	0.084	0.189	2.680 **			
Step 4				0.63	0.04	56.06 **
Gender	0.766	0.052	0.919			
DS-IT	0.398	−0.796	−12.35 **			
PDI	0.082	0.184	2.853 *			
Positive religious coping	0.381	0.291	5.199 **			
Step 5				0.67	0.66	53.36 **
Gender	0.815	0.055	1.034			
DS-IT	−0.364	−0.727	−11.467 **			
PDI	0.056	0.127	2.021 *			
Positive religious coping	0.195	0.148	2.341 *			
TCI-ST	0.105	0.256	4.069 **			

Abbreviations. FACIT-Sp-12: Functional Assessment of Chronic Illness Therapy—Spiritual Wellbeing Scale; DS-IT: Demoralization Scale, Italian Version; PDI: Patient Dignity Inventory; TCI-ST: Temperament and Character Inventory—Self-Transcendence Scale. * *p* value < 0.05; ** *p* value < 0.001.

## Data Availability

All relevant data are contained within the manuscript file. The dataset used and/or analyzed during the current study is available from the corresponding author upon reasonable request.

## References

[1] Steinhorn D.M., Din J., Johnson A. (2017). Healing, spirituality and integrative medicine. Ann. Palliat. Med..

[2] Scheffold K., Philipp R., Vehling S., Koranyi S., Engelmann D., Schulz-Kindermann F., Härter M., Mehnert-Theuerkauf A. (2019). Spiritual well-being mediates the association between attachment insecurity and psychological distress in advanced cancer patients. Support. Care Cancer.

[3] Santos P.R., Júnior J.R.F.G.C., Filho J.R.M.C., Ferreira T.P., Oliveira S.D.S. (2017). Religious coping methods predict depression and quality of life among end-stage renal disease patients undergoing hemodialysis: A cross-sectional study. BMC Nephrol..

[4] Tarakeshwar N., Vanderwerker L.C., Paulk E., Pearce M.J., Kasl S.V., Prigerson H.G., King S.D., Macpherson C.F., Pflugeisen B.M., Johnson R.H. (2006). Religious Coping is Associated with the Quality of Life of Patients with Advanced Cancer. J. Palliat. Med..

[5] Zamanian H., Eftekhar-Ardebili H., Eftekhar-Ardebili M., Shojaeizadeh D., Nedjat S., Taheri-Kharameh Z., Daryaafzoon M. (2015). Religious Coping and Quality of Life in Women with Breast Cancer. Asian Pac. J. Cancer Prev..

[6] Haghighi F. (2013). Correlation between religious coping and depression in cancer patients. Psychiatr. Danub..

[7] Karabulutlu E.Y., Yaralı S., Karaman S. (2019). Evaluation of Distress and Religious Coping Among Cancer Patients in Turkey. J. Relig. Health.

[8] Pargament K.I., Koenig H.G., Perez L.M. (2000). The many methods of religious coping: Development and initial validation of the RCOPE. J. Clin. Psychol..

[9] Krok D. (2014). The Role of Meaning in Life Within the Relations of Religious Coping and Psychological Well-Being. J. Relig. Health.

[10] Coward D.D., Reed P.G. (1996). Self-Transcendence: A Resource for Healing at the End of Life. Issues Ment. Health Nurs..

[11] Monasterio E., Cloninger C.R. (2019). Self-Transcendence in Mountaineering and BASE Jumping. Front. Psychol..

[12] Cloninger C.R., Svrakic D.M., Przybeck T.R. (1993). A Psychobiological Model of Temperament and Character. Arch. Gen. Psychiatry.

[13] Levenson M.R., Jennings P.A., Aldwin C.M., Shiraishi R.W. (2005). Self-Transcendence: Conceptualization and Measurement. Int. J. Aging Hum. Dev..

[14] Guerrero-Castañeda R.F., Menezes T.M.D.O., Prado M.L.D., Galindo-Soto J.A. (2019). Spirituality and religiosity for the transcendence of the elderly being. Rev. Bras. de Enferm..

[15] Haugan G., Moksnes U.K., Løhre A. (2016). Intrapersonal self-transcendence, meaning-in-life and nurse-patient interaction: Powerful assets for quality of life in cognitively intact nursing-home patients. Scand. J. Caring Sci..

[16] Chaar E.A., Hallit S., Hajj A., Aaraj R., Kattan J., Jabbour H., Khabbaz L.R. (2018). Evaluating the impact of spirituality on the quality of life, anxiety, and depression among patients with cancer: An observational transversal study. Support. Care Cancer.

[17] Reed P.G. (2008). Theory of self-transcendence. Middle Range Theory Nurs..

[18] Vehling S., Lehmann C., Oechsle K., Bokemeyer C., Krüll A., Koch U., Mehnert A. (2011). Global meaning and meaning-related life attitudes: Exploring their role in predicting depression, anxiety, and demoralization in cancer patients. Support. Care Cancer.

[19] Greenway A.P., Phelan M., Turnbull S., Milne L.C. (2007). Religious coping strategies and spiritual transcendence. Ment. Health Relig. Cult..

[20] Pargament K.I., Smith B.W., Koenig H.G., Perez L. (1998). Patterns of Positive and Negative Religious Coping with Major Life Stressors. J. Sci. Study Relig..

[21] Ripamonti C.I., Giuntoli F., Gonella S., Miccinesi G. (2018). Spiritual care in cancer patients: A need or an option?. Curr. Opin. Oncol..

[22] Sharif S.P., Lehto R.H., Nia H.S., Goudarzian A.H., Haghdoost A.A., Yaghoobzadeh A., Tahmasbi B., Nazari R. (2018). Religious coping and death depression in Iranian patients with cancer: Relationships to disease stage. Support. Care Cancer.

[23] Sun V., Kim J.Y., Irish T.L., Borneman T., Sidhu R.K., Klein L., Ferrell B. (2016). Palliative care and spiritual well-being in lung cancer patients and family caregivers. Psycho-Oncol..

[24] Gijsberts M.-J.H.E., Liefbroer A.I., Otten R., Olsman E. (2019). Spiritual Care in Palliative Care: A Systematic Review of the Recent European Literature. Med. Sci..

[25] Best M.C., Vivat B., Gijsberts M.-J. (2023). Spiritual Care in Palliative Care. Religions.

[26] Thomas J.C., Burton M., Griffin M.T.Q., Fitzpatrick J.J. (2010). Self-Transcendence, Spiritual Well-Being, and Spiritual Practices of Women With Breast Cancer. J. Holist. Nurs..

[27] Carlson L.E., Zelinski E.L., Toivonen K.I., Sundstrom L., Jobin C.T., Damaskos P., Zebrack B. (2019). Prevalence of psychosocial distress in cancer patients across 55 North American cancer centers. J. Psychosoc. Oncol..

[28] Pandey M., Sarita G.P., Devi N., Thomas B.C., Hussain B.M., Krishnan R. (2006). Distress, anxiety, and depression in cancer patients undergoing chemotherapy. World J. Surg. Oncol..

[29] Brintzenhofe-Szoc K.M., Levin T.T., Li Y., Kissane D.W., Zabora J.R. (2009). Mixed Anxiety/Depression Symptoms in a Large Cancer Cohort: Prevalence by Cancer Type. Psychosomatics.

[30] Linden W., Vodermaier A., MacKenzie R., Greig D. (2012). Anxiety and depression after cancer diagnosis: Prevalence rates by cancer type, gender, and age. J. Affect. Disord..

[31] Angelino A.F., Treisman G.J. (2001). Major depression and demoralization in cancer patients: Diagnostic and treatment considerations. Support. Care Cancer.

[32] Mehnert A., Vehling S., Höcker A., Lehmann C., Koch U. (2011). Demoralization and Depression in Patients With Advanced Cancer: Validation of the German Version of the Demoralization Scale. J. Pain Symptom Manag..

[33] Chochinov H.M., Krisjanson L.J., Hack T.F., Hassard T., McClement S., Harlos M. (2006). Dignity in the Terminally Ill: Revisited. J. Palliat. Med..

[34] Vehling S., Mehnert A. (2014). Symptom burden, loss of dignity, and demoralization in patients with cancer: A mediation model. Psycho-Oncol..

[35] Karnofsky D., Burchenal J.H. (1949). Evaluation of Chemotherapeutic Agents.

[36] Franchini L., Ercolani G., Ostan R., Raccichini M., Samolsky-Dekel A., Malerba M., Melis A., Varani S., Pannuti R. (2019). Caregivers in home palliative care: Gender, psychological aspects, and patient’s functional status as main predictors for their quality of life. Support. Care Cancer.

[37] Schag C.C., Heinrich R.L., Ganz P.A. (1984). Karnofsky performance status revisited: Reliability, validity, and guidelines. J. Clin. Oncol..

[38] Peterman A.H., Fitchett G., Brady M.J., Hernandez L., Cella D. (2002). Measuring spiritual well-being in people with cancer: The functional assessment of chronic illness therapy—Spiritual well-being scale (FACIT-Sp). Ann. Behav. Med..

[39] Essue B.M., Iragorri N., Ma N.F., De Oliveira C. (1999). The psychosocial cost burden of cancer: A systematic literature review. Psycho-Oncol..

[40] Rabitti E., Cavuto S., Iani L., Ottonelli S., De Vincenzo F., Costantini M. (2020). The assessment of spiritual well-being in cancer patients with advanced disease: Which are its meaningful dimensions?. BMC Palliat. Care.

[41] Cloninger C.R. (1999). The Temperament and Character Inventory-Revised.

[42] Fossati A., Cloninger C.R., Villa D., Borroni S., Grazioli F., Giarolli L., Battaglia M., Maffei C. (2007). Reliability and validity of the Italian version of the Temperament and Character Inventory-Revised in an outpatient sample. Compr. Psychiatry.

[43] Farmer R.F., Goldberg L.R. (2008). A psychometric evaluation of the revised Temperament and Character Inventory (TCI-R) and the TCI-140. Psychol. Assess..

[44] Garcia-Romeu A. (2010). Self-transcendence as a measurable transpersonal construct. J. Transpers. Psychol..

[45] Pargament K., Feuille M., Burdzy D. (2011). The Brief RCOPE: Current Psychometric Status of a Short Measure of Religious Coping. Religions.

[46] Giaquinto S., Cipolla F., Giachetti I., Onorati D. (2011). Italian validation of the Brief Rcope scale for religious coping. J. Med. Pers..

[47] Chochinov H.M., Hassard T., McClement S., Hack T., Kristjanson L.J., Harlos M., Sinclair S., Murray A. (2008). The Patient Dignity Inventory: A Novel Way of Measuring Dignity-Related Distress in Palliative Care. J. Pain Symptom Manag..

[48] Ripamonti C.I., Buonaccorso L., Maruelli A., Bandieri E., Pessi M.A., Boldini S., Primi C., Miccinesi G. (2012). Patient dignity inventory (PDI) questionnaire: The validation study in Italian patients with solid and hematological cancers on active oncological treatments. Tumori J..

[49] Chochinov H.M., McClement S.E., Hack T.F., McKeen N.A., Rach A.M., Gagnon P., Sinclair S., Taylor-Brown J. (2012). The Patient Dignity Inventory: Applications in the Oncology Setting. J. Palliat. Med..

[50] Kissane D.W., Wein S., Love A., Lee X.Q., Kee P.L., Clarke D.M. (2004). The Demoralization Scale: A report of its de-velopment and preliminary validation. J. Palliat. Care.

[51] Costantini A., Picardi A., Brunetti S., Trabucchi G., Bersani F.S., Minichino A., Marchetti P. (2013). La versione italiana della Demoralization Scale: Uno studio di validazione. Riv. Psichiatr..

[52] Van Cappellen P., Saroglou V., Iweins C., Piovesana M., Fredrickson B.L. (2013). Self-transcendent positive emotions increase spirituality through basic world assumptions. Cogn. Emot..

[53] Aydın A., Işık A., Kahraman N. (2020). Mental health symptoms, spiritual well-being and meaning in life among older adults living in nursing homes and community dwellings. Psychogeriatrics.

[54] Bernard M., Strasser F., Gamondi C., Braunschweig G., Forster M., Kaspers-Elekes K., Veri S.W., Borasio G.D., Pralong G., Pralong J. (2017). Relationship Between Spirituality, Meaning in Life, Psychological Distress, Wish for Hastened Death, and Their Influence on Quality of Life in Palliative Care Patients. J. Pain Symptom Manag..

[55] Kirk K.M., Eaves L.J., Martin N.G. (1999). Self-transcendence as a measure of spirituality in a sample of older Australian twins. Twin Res. Hum. Genet..

[56] Frankl V.E. (1967). Logotherapy and existentialism. Psychotherapy.

[57] Breitbart W., Gibson C., Poppito S.R., Berg A. (2004). Psychotherapeutic Interventions at the End of Life: A Focus on Meaning and Spirituality. Can. J. Psychiatry.

[58] Chochinov H.M., Hack T., Hassard T., Kristjanson L.J., McClement S., Harlos M. (2005). Dignity Therapy: A Novel Psychotherapeutic Intervention for Patients Near the End of Life. J. Clin. Oncol..

[59] Fitchett G., Emanuel L., Handzo G., Boyken L., Wilkie D.J. (2015). Care of the human spirit and the role of dignity therapy: A systematic review of dignity therapy research. BMC Palliat. Care.

[60] Timmins F., Caldeira S., Murphy M., Pujol N., Sheaf G., Weathers E., Whelan J., Flanagan B. (2018). The Role of the Healthcare Chaplain: A Literature Review. J. Health Care Chaplain..

[61] Saracino R.M., Rosenfeld B., Breitbart W., Chochinov H.M. (2019). Psychotherapy at the End of Life. Am. J. Bioeth..

